# Supplementary material for: Lysosomal ATP Transporter SLC17A9 Controls Cell Viability via Regulating Cathepsin D
Source: Cells. 2022 Mar 4;11(5):887. doi: 10.3390/cells11050887 (PMC8909234; doi:10.3390/cells11050887)
Supplement: Supplementary file 1 [file cells-11-00887-s001.zip › cells-1378021-supplementary.pdf]

cells

MDPI

Article

Lysosomal ATP Transporter SLC17A9 Controls Cell Viability via Regulating Cathepsin D

Peng Huang[1,2,3†], Qi Cao[3,†], Mengnan Xu[3], and Xian-Ping Dong[3,*]

1 School of Clinical Medicine, Shanghai University of Medicine and Health Sciences, Shanghai 201318, China
2 Collaborative Innovation Center for Biomedicine, Shanghai University of Medicine and Health Sciences, Shanghai 201318, China
3 Department of Physiology and Biophysics, Dalhousie University, Sir Charles Tupper Medical Building, 5850 College Street, Halifax, B3H 4R2, Nova Scotia, Canada
* Correspondence: xpdong@dal.ca
† Equal contributions.

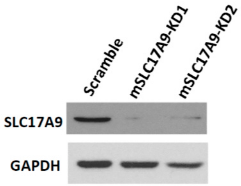

Figure S1. Deletion of mSLC17A9 by mSLC17A9 shRNAs. Whole cell lysates were used, and GAPDH levels served as loading controls.

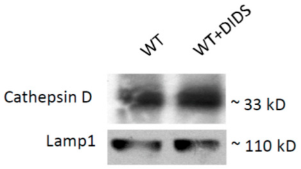

Figure S2. DIDS treatment did not decrease Cathepsin D expression in lysosomes of Cos1 cells. The 34 kDa lysosomal (mature) forms of cathepsin D were shown. Lamp1 was blotted show a comparable number of lysosomes between two groups.

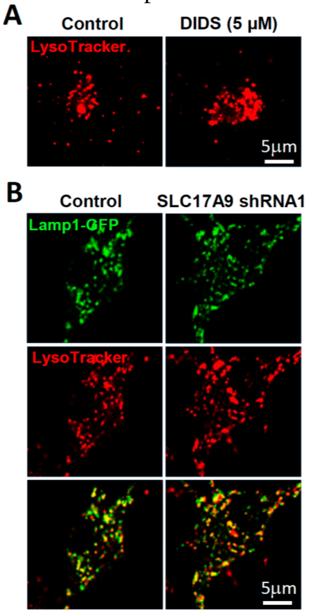

Figure S3. Lysosomal acidification was not affected by SLC17A9 deficiency. (A) DIDS (5 μM, 6 hrs) treatment did not alter LysoTracker signals in Cos1 cells. (B) LysoTracker signals was not affected by SLC17A9 knockdown in C2C12 cells.
